# The Clinical Significance of MiR-148a as a Predictive Biomarker in Patients with Advanced Colorectal Cancer

**DOI:** 10.1371/journal.pone.0046684

**Published:** 2012-10-03

**Authors:** Masanobu Takahashi, Miriam Cuatrecasas, Francesc Balaguer, Keun Hur, Yuji Toiyama, Antoni Castells, C. Richard Boland, Ajay Goel

**Affiliations:** 1 Gastrointestinal Cancer Research Laboratory, Baylor Research Institute and Charles A Sammons Cancer Center, Baylor University Medical Center, Dallas, Texas, United States of America; 2 Pathology Department, Centro de Diagnóstico Biomédico (CDB), Tumour Bank, Hospital Clínic, Institut d'Investigacions Biomèdiques August Pi i Sunyer (IDIBAPS) Biobank Hospital Clínic, University of Barcelona, Barcelona, Spain; 3 Gastroenterology Department, Hospital Clínic, Centro de Investigación Biomédica en Red de Enfermedades Hepáticas y Digestivas (CIBEREHD), IDIBAPS, University of Barcelona, Barcelona, Spain; University of Barcelona, Spain

## Abstract

**Aim:**

Development of robust prognostic and/or predictive biomarkers in patients with colorectal cancer (CRC) is imperative for advancing treatment strategies for this disease. We aimed to determine whether expression status of certain miRNAs might have prognostic/predictive value in CRC patients treated with conventional cytotoxic chemotherapies.

**Methods:**

We studied a cohort of 273 CRC specimens from stage II/III patients treated with 5-fluorouracil-based adjuvant chemotherapy and stage IV patients subjected to 5-fluorouracil and oxaliplatin-based chemotherapy. In a screening set (n = 44), 13 of 21 candidate miRNAs were successfully quantified by multiplex quantitative RT-PCR. In the validation set comprising of the entire patient cohort, miR-148a expression status was assessed by quantitative RT-PCR, and its promoter methylation was quantified by bisulfite pyrosequencing. Lastly, we analyzed the associations between miR-148a expression and patient survival.

**Results:**

Among the candidate miRNAs studied, miR-148a expression was most significantly down-regulated in advanced CRC tissues. In stage III and IV CRC, low miR-148a expression was associated with significantly shorter disease free-survival (DFS), a worse therapeutic response, and poor overall survival (OS). Furthermore, miR-148a methylation status correlated inversely with its expression, and was associated with worse survival in stage IV CRC. In multivariate analysis, miR-148a expression was an independent prognostic/predictive biomarker for advanced CRC patients (DFS in stage III, low vs. high expression, HR 2.11; OS in stage IV, HR 1.93).

**Discussion:**

MiR-148a status has a prognostic/predictive value in advanced CRC patients treated with conventional chemotherapy, which has important clinical implications in improving therapeutic strategies and personalized management of this malignancy.

## Introduction

Currently, colorectal cancer (CRC) patients with lymph node metastasis (TNM stage III) are treated with adjuvant chemotherapy that includes cytotoxic drugs such as 5-fluorouracil (5-FU) and oxaliplatin, following surgical resection of the cancer. Similarly, patients with distant metastatic CRC (stage IV) are treated with various combinations of chemotherapeutic drugs and molecularly-targeted drugs that include anti-VEGF and anti-EGFR antibodies. Although these treatment regimens have improved outcomes in patients with advanced CRC, a significant proportion of individuals fail to derive any benefit from such treatments, and some experience worse outcomes as a result of drug-associated toxicities. There is an imperative need for developing predictive biomarkers that can select the subgroup of patients that will benefit from conventional chemotherapeutic drugs, so that patients who will not benefit from the treatment can be spared from drug toxicity and offered alternate treatments.

Mutations in *KRAS* are the only established predictive markers for selecting treatment strategies in CRC. Patients with tumors harboring mutations in codon 12 or 13 of the *KRAS* gene do not benefit from anti-EGFR-based drug therapy [1], [2], and consequently, screening for this mutation is recommended for all stage IV patients who are candidates for anti-EGFR antibody-based drug therapy. In addition, microsatellite instability (MSI), a phenotype present in ∼15% of CRCs, has been shown to associate with improved overall survival (OS) regardless of adjuvant chemotherapy, as well as a lack of benefit from 5-FU-based chemotherapy in stage II patients [3], [4]. However, whether MSI has predictive value in stage III patients treated with chemotherapy remains uncertain [5], [6]. While some studies have suggested that other molecular markers, such as the CpG island methylator phenotype or expression status of genes involved in DNA repair or drug metabolism (such as *ERCC1* and *TYMS*) may have potential as prognostic/predictive markers, there is insufficient consistent data supporting the usefulness of these markers [7]–[11]. Taken together, in spite of strides made in the genomic and epigenomic characterization of CRC, there are no *established* biomarkers that can reliably predict therapeutic outcomes from conventional cytotoxic chemotherapy in patients with stage III or IV CRC.

Dysregulation of microRNA (miRNA) expression is implicated in early tumorigenesis as well as disease progression in various malignancies [12], [13]. miRNAs exert their oncogenic and/or tumor suppressive effects by binding to the 3′-untranslated regions of mRNA, leading to suppression of translation or enhanced degradation of the corresponding message. In CRC, dysregulated expression of several miRNAs, including the miR-17∼92, miR-21, miR-31, miR-34b/c, miR-143, miR-145, and miR-203 have been previously reported [14]–[17]. Since miRNAs play a central role in human carcinogenesis, there is growing interest in the identification of prognostic or predictive miRNAs in patients with CRC.

However, only a handful of reports have investigated the potential of miRNA(s) as prognostic/predictive biomarkers in CRC. In a seminal study, Schetter *et al.* demonstrated that miR-21 may be a promising prognostic/predictive marker in stage II/III CRC treated with 5-FU-based chemotherapy [17]. Similarly, another group has shown that stage II patients with elevated expression of miR-320 or miR-498 had better recurrence-free survivals [18]. However, the sample sizes analyzed in these studies were relatively small, the results have not been validated in other studies, and there has been no attempt to determine the predictive utility of any miRNA in stage IV CRC. We hypothesized that the expression profiles of specific miRNA(s) might have prognostic and/or predictive value in patients with stage IV CRC as well as those with earlier stages. Herein, in an initial screening step that involved analysis of 21candidate miRNAs, we discovered that miR-148a is frequently downregulated in advanced CRC. Subsequently, by analyzing a large validation cohort of 273 CRCs, we provide novel evidence that miR-148a is frequently down-regulated in this disease, and this principally occurs through hypermethylation of its putative promoter region. In addition, we demonstrate that low miR-148a expression is significantly associated with an unfavorable outcome in patients with stage III CRC treated with 5-FU-based chemotherapy and with a worse therapeutic response and survival in patients with stage IV CRC treated with 5-FU and oxaliplatin.

## Patients and Methods

### Ethics Statement

The study was approved by the Institutional Review Boards (IRB) of Hospital Clinic, Barcelona, Spain, and a written informed consent was obtained from all patients.

### Patients

Formalin-fixed paraffin-embedded (FFPE) tissues from a cohort of 273 patients with CRC (primary tumors from 76 stage II, 125 stage III, and 72 stage IV CRCs) and 20 tissue specimens from normal colonic mucosa of healthy individuals were obtained from the Pathology Department of the Hospital Clinic, Barcelona, Spain. The patients included in this study were enrolled between 1996 and 2008. All stage II and III patients were treated with 5-FU-based adjuvant chemotherapy for 6 months subsequent to tumor resection, and all stage IV patients were treated with 5-FU and oxaliplatin until the treatment failed. The stage II and III patients were followed-up every three months for the first two years, and every six months for the subsequent three years. Both locoregional relapse and/or distant metastasis were defined as tumor recurrence, whereas metachronous colorectal lesions were not considered as recurrence. The median follow-up times are 52.2 months (range; 2.9–173 months) in stage II and III patients, and 19.1 months (range; 3.7–83.7 months) in stage IV patients. Among stage II and III patients, 70 patients (35%) had tumor recurrence (median; 17.8 months, range: 5.5–144 months), and the median DFS of non-recurrence patients were 40.5 months (range; 7.5–155 months). The follow-up of patients was finished in November, 2009. Chemotherapeutic response in stage IV patients was evaluated according to the Response Evaluation Criteria In Solid Tumors (RECIST) guidelines [19] every two months. MSI status of tumors was determined by analyzing five mononucleotide markers (BAT-25, BAT-26, MONO-27, NR-21, and NR-24; MSI Analysis System, Promega, Madison, Wisconsin, USA). The clinicopathological characteristics of the patients are shown in **Table 1**.

**Table 1:** MiR-148 expression status and clinicopathologic characteristics of CRC patients.

		Stage II+III					Stage IV				
		High expression		Low expression			High expression		Low Expression		
		(n = 138)		(n = 63)			(n = 36)		(n = 36)		
		N	%	N	%	p	N	%	N	%	p
**Age, years**	Median	66.5		68.5		0.13^a^	58.5		62.0		0.36^a^
	Range	32–82		45–82			43–78		36–79		
**Gender**	Male	79	57	39	62	0.64^b^	25	69	22	61	0.62^b^
	Female	59	43	24	38		11	31	14	39	
**Tumors location** c	Proximal	39	28	22	35	0.41^d^	10	28	5	14	0.35^d^
	Distal	99	72	41	65		20	56	24	67	
	Rectum	0	0	0	0		6	17	7	19	
**MSI**	Yes	8	6	6	10	0.38^b^	0	0	1	3	1.00^b^
	No	130	94	57	90		36	100	35	97	
**Performance status**	0–1						32	89	31	86	1.00^b^
	2						4	11	5	14	
**Tumor response**	CR+PR						29	81	17	47	0.006^b^
	SD+PD						7	19	19	53	

a The difference was analyzed by Mann-Whitney U test.

b The difference was analyzed by Fisher's exact test.

c Proximal colon, located above splenic flexure; distal colon, located in splenic flexure or below.

d The difference was analyzed by the chi-square test.

### DNA and RNA extraction

DNA was extracted from 10 µm-thick FFPE tissues using the QIAmp DNA FFPE tissue kit (Qiagen, Valencia, California, USA). Total RNA including the miRNA fraction was extracted from FFPE tissues using the RecoverAll Total Nucleic Acid Isolation Kit (Ambion, Inc., Austin, Texas, USA).

### Multiplex quantitative RT-PCR

In a screening set that included normal colonic mucosa from healthy subjects and 44 CRC tissues (16 stage II and III each and 12 stage IV patients), the expression status of 21 candidate miRNAs (miR-9, miR-10b, miR-19a, miR-21, miR-31, miR-34a, miR-34c, miR-101, miR-103, miR-137, miR-143, miR-145, miR-148a, miR-148b, miR-152, miR-155, miR-194, miR-320, miR-335, miR-373 and miR-519c) was quantified using the high-throughput Fluidigm microfluidics dynamic arrays [20]. Each Taqman miRNA assay (part no. 4427975, Applied Biosystems, Foster City, California, USA) was used in the multiplex RT-PCR analysis as follows: assay ID, 000583, 002218, 000395, 000397, 002279, 000426, 000428, 002253, 000439, 001129, 002249, 002278, 000470, 000471, 000475, 002623, 000493, 002277, 000546, 000561, and 001163, respectively. These candidate miRNAs have previously been shown to be involved in CRC and/or other human malignancies [16], [18], [21]–[35].

### Quantification of miRNA expression by real-time RT-PCR

In the validation set that included the entire patient cohort, miRNA expression was quantified by Taqman reverse transcription-PCR (qRT-PCR) using an ABI 7000 sequence detection system (Applied Biosystems). The expression of miR-148a was calculated by the delta Ct value method, using miR-16 expression as a normalizer [36], [37]. To keep consistent measurements throughout all plates, three independent RNA samples were loaded as internal controls in each PCR run, and the results from each plate were normalized according to data obtained from internal controls.

### DNA methylation analysis

DNA was bisulfite modified using the EZ DNA methylation Gold Kit (Zymo Research, Irvine, California, USA). Methylation of putative miR-148a promoter region was quantified by bisulfite pyrosequencing (PSQ HS 96A pyrosequencing system, Qiagen). The following primers were used; miR-148a forward, 5′-biotin-TAGGAAGGAAGGAGAGTG, miR-148a reverse, 5′-CCCAACAAAAATAATATTTTAACA, and miR-148a sequencing, 5′-CAAAAATAATATTTTAACAACC. The methylation levels of three CpG sites were analyzed and the methylation level of each tumor is represented as the mean value of methylation levels of the three CpG sites. The following PCR cycle conditions were used: initial denaturation at 94°C for 7 min, followed by 45 cycles at 94°C for 30 sec, 52°C for 30 sec, and 72°C for 30 sec.

### *In situ* hybridization for miR-148a

*In situ* hybridization (ISH) was performed as described by Navarro et al. [38] with minor adjustments. A fluorescein (FITC) 5′-labeled locked nucleic acid-incorporated miRNA probe (miRCURY LNA detection probe, Exiqon, Woburn, Massachusetts, USA) was used for visualization of miR-148a on 3 µm-thick FFPE tissue sections. A scrambled and an RNU6b probe were included as negative and positive controls, respectively (Exiqon). The slides were placed in an oven at 59°C overnight. Sections were deparaffinized with xylene, rehydrated with ethanol, and treated with diethylpyrocarbonate water for 1 min. Chromogenic ISH was performed in an automated platform Bond Max (Vision BioSystems, Norwell, Massachusetts, USA). Slides were pretreated with protease 1 for 4 min at 37°C. A total of 300 µl 25-nM probe was hybridized in sodium chloride, sodium citrate hybridization buffer at 45°C overnight. Immunologic detection was performed with a mouse anti-FITC antibody at 37°C for 60 min followed by a biotin-free, polymeric horseradish peroxidase linker antibody conjugate system (Refine Detection System, Vision BioSystems). DAB was used as the chromogen and hematoxylin was used as a counterstain.

### Statistical analysis

Statistical analyses were performed with GraphPad Prism 4.0 (GraphPad Software, La Jolla, California, USA) or MedCalc v12 (MedCalc software, Belgium). The differences between two groups were analyzed by the Mann-Whitney U-test. Correlation analyses were carried out using Spearman's rank correlation method. The CRC tumors were categorized into high and low miR-148 expression groups using Receiver Operating Characteristic curve analysis (stage II/III) or the median expression values (stage IV). Kaplan-Meier analysis was performed to estimate the distributions of disease-free survival (DFS) and cancer-specific overall survival (OS) in stage II and III patients, and progression-free survival (PFS) and OS in stage IV patients. A log-rank test was used to analyze the statistical differences in survival as deduced from Kaplan-Meier curves. Cox proportional-hazard regression analysis was performed to calculate HR and 95% CI for each covariable. The final multivariate model was based upon a stepwise method for clinical factors associated with good or poor survival (p<0.1) in univariate models. For the survival analysis, the solitary MSI tumor was excluded from the stage IV group. All differences were regarded as statistically significant when p<0.05.

## Results

### miR-148a is a candidate miRNA which is frequently down-regulated in stage III and IV CRC

In a screening set, we first screened 21 candidate miRNAs that have been implicated in tumorigenesis, using the Fluidigm microfluidics dynamic arrays (n = 44; **Supplementary Table S1**). Among the 21 miRNAs analyzed, 13 miRNA successfully yielded expression results in the multiplex RT-PCR. Seven miRNAs demonstrated significantly altered expression between normal and CRC tissues. Among all, only miR-148a revealed a significant down-regulation in tumors with lymph node (stage III) or distant metastasis (IV) compared to tumors without (II), which underscores the significance of this miRNA in CRC progression/metastasis. Based upon these results, we subsequently determined whether a miR-148a expression alteration in a larger cohort of CRC has any prognostic/predictive value.

**Figure 1:**
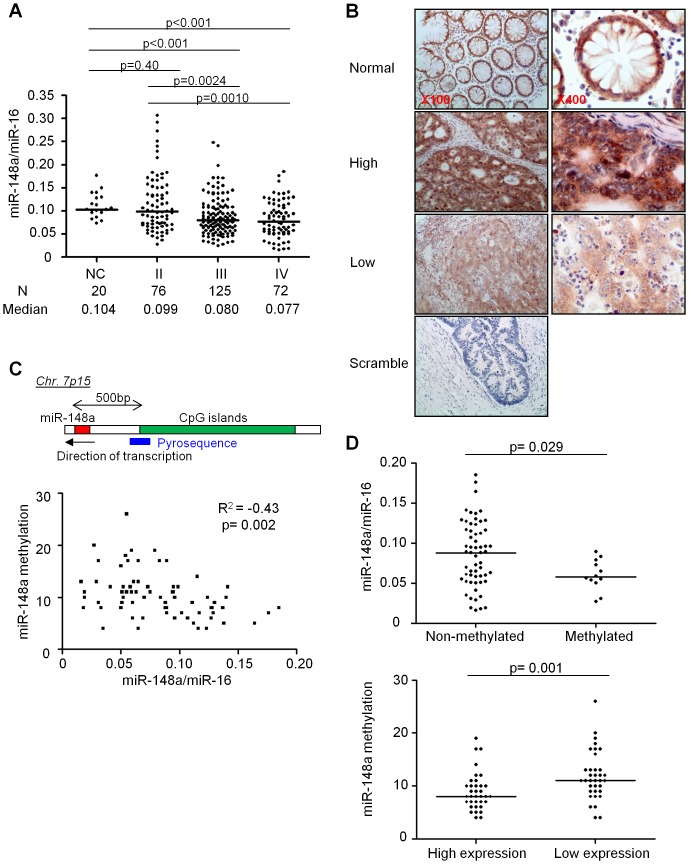
MiR-148a expression and methylation in colonic mucosa from healthy individuals and in CRC tissues from patients. **A**. miR-148a expression in colonic mucosa from healthy controls (NC), and in stage II, III and IV CRCs; the number of patients (N) and median expression (Median) are listed below the graph. **B**. *In situ* hybridization for miR-148a in CRC tumors and normal mucosa, in which the chromogen stains red, and the counterstain blue. Representative photomicrographs are shown from a normal colonic mucosa (top panels), a tumor with low miR-148a expression (middle panels), and a tumor with high miR-148a expression (lower panels) at indicated magnifications. A photomicrograph is shown from a tumor with high miR-148a expression using a scramble probe as a negative control (bottom, left panel). **C**. miR-148a methylation levels in stage IV tumors. The putative promoter region of miR-148a, and the position of pyrosequencing primers are illustrated in the top panel. The scatter plot of miR-148a expression and methylation levels are shown in the bottom panel. **D**. miR-148a expression levels are shown for methylated and non-methylated CRCs in the top panel. miR-148a methylation levels are shown for tumors with high and low miR-148a expression in the bottom panel. One outlier value (the methylation level; 48%) is excluded from the methylated group in the bottom graph.

In our entire cohort (n = 273), miR-148a expression in stage III/IV tumors was significantly lower than in normal colonic mucosa (p<0.001; **Figure 1A**). We also observed a trend toward gradual lowering of miR-148a expression with advancing stage of the CRCs (**Figure 1A**). More specifically, miR-148a expression in stage III and IV tumors was significantly lower than in normal colonic mucosa (p<0.001), while it was not significantly different between stage II tumors and the normal mucosal specimens (p = 0.41; **Figure 1A**).

Next, to confirm the tumor-specific expression pattern for miR-148a, we performed ISH analysis in a subset of stage IV tumors with high and low miR-148a expression. We observed that expression in normal colonic mucosa of stage II tumors was high, confirming the qRT-PCR results (**Figure 1B**). CRCs with high miR-148a expression at qRT-PCR also expressed this miRNA primarily within the cytoplasm of neoplastic cells (**Figure 1B**), but not in the non-epithelial stromal cells, except for the staining of some inflammatory cells in the lamina propria, particularly the plasma cells. Furthermore, CRCs with low miR-148a expression ascertained by qRT-PCR also revealed very low or absent expression of this miRNA at the ISH level (**Figure 1B**). These results indicate that our qRT-PCR results accurately reflected the endogenous expression of miR-148a within the cancer cells obtained from CRC tissue specimens.

### Expression of miR-148a is inversely correlated with its promoter methylation status

The putative promoter region of miR-148a sites within a CpG island, and methylation of these CpG sites have been proposed as a potential mechanism for miR-148a inactivation in CRC and breast cancers [39]–[41]. However, none of previous studies have thoroughly investigated the direct correlation between miR-148a expression and its methylation status in a large cohort of cancer specimens. Accordingly, we were interested in elucidating whether the down-regulation of miR-148a observed in our cohort was a consequence of promoter hypermethylation. Since miR-148a was most frequently down-regulated in stage IV CRC, we focused our methylation analysis on these tumors. Quantitative bisulfite pyrosequencing revealed that some stage IV CRCs demonstrated miR-148a hypermethylation. The methylation levels ranged between 4–26% (median, 10%), and when the methylation status of each tumor was compared with its qRT-PCR-derived expression status, a significant correlation was observed (Spearman's coefficient, R^2^ = −0.43, p<0.001; **Figure 1C**). Additionally, when we categorized all tumors into a non-methylated (methylation level <15%) and methylated groups (≥15% methylation), we observed that the methylated tumors had consistently lower miR-148a expression (0.068 vs. 0.088, p = 0.029; **Figure 1D** **, top**). Furthermore, CRCs with lower miR-148a expression were more frequently methylated compared to tumors with higher expression (median, 11% vs. 8%, p = 0.001, Mann-Whitney U test; **Figure 1D** **, bottom**). These results highlight that the hypermethylation of the putative miR-148a promoter region is an important regulatory mechanism for its expression in CRC.

### Low miR-148a expression is associated with poor outcome in patients with stage II and III CRC

**Figure 2:**
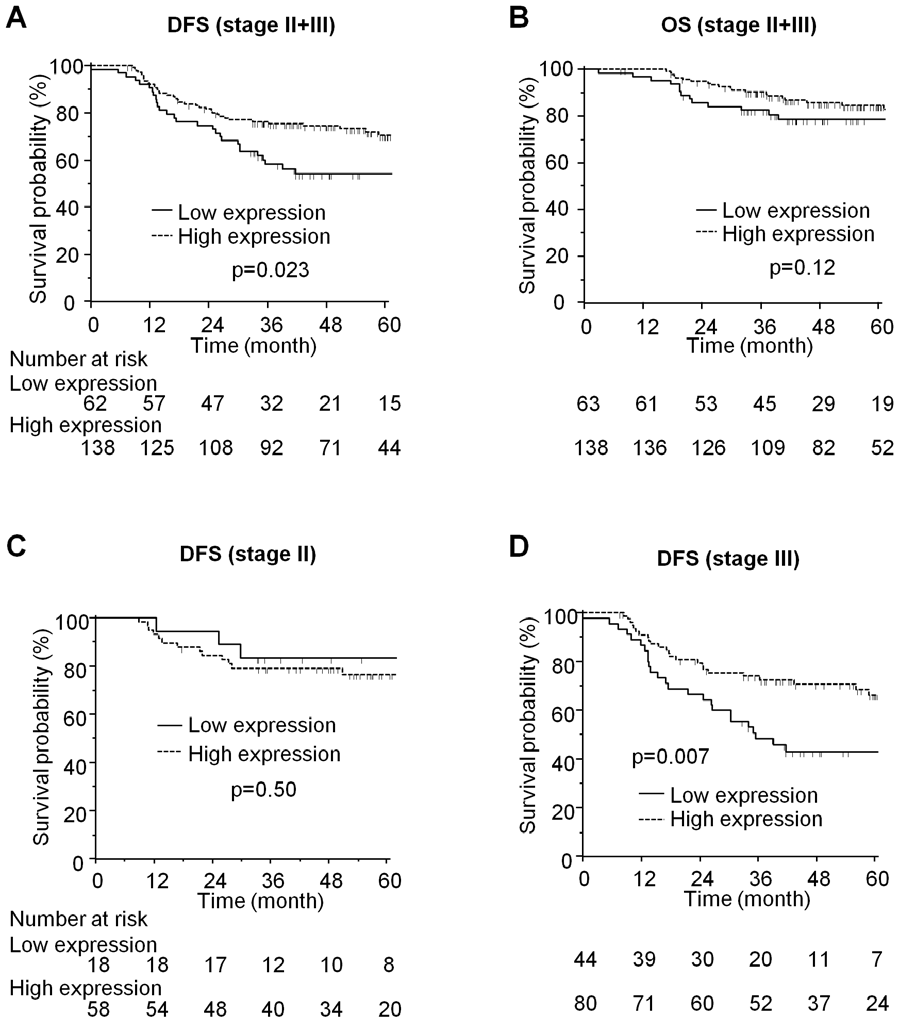
Survival analysis in stage II/III patients treated with 5-FU-based chemotherapy. Kaplan-Meyer curves for **A**, Disease-free survival (DFS) and **B**, overall survival (OS) in stage II/III patients according to miR-148a expression. Kaplan-Meyer curves for **C**, DFS in stage II and **D**, stage III DFS depending upon miR-148a expression.

We next aimed to determine whether miR-148a expression status had an impact on prognosis in patients with stage II and III CRC treated with 5-FU-based adjuvant chemotherapy. For these analyses, we compared the differences in DFS and OS between the high expression (stage II = 58, III = 80) and low expression (II = 18, III = 45) groups. We did not find significant associations between the miR-148a expression and any of the clinicopathological factors such as age, gender, tumor location or MSI status (**Table 1**). However, low miR-148a expression was significantly associated with shorter DFS (5-year DFS, low vs. high, 54% vs. 71%, p = 0.023; **Figure 2A**), and showed a trend toward worse OS (5-year OS, 78% vs. 85%, p = 0.12; **Figure 2B**). We next evaluated the prognostic/predictive value of miR-148a expression in a Cox proportional hazard regression model. In univariate analysis, higher TNM stage (III vs. II, HR 2.06, 95% CI 1.21–3.52, p = 0.008) and lower miR-148a expression (HR 1.74, 95% CI 1.08–2.83, p = 0.025) were significantly associated with shorter DFS, and younger age showed a trend towards shorter DFS (<60, HR 1.57, 95% CI 0.97–2.56, p = 0.071; **Table 2**). Furthermore, in the multivariate model including these three factors, miR-148a expression status was independently associated with worse survival (HR 1.83, 95% CI 1.12–2.99, p = 0.017; **Table 2**).

**Table 2:** Univariate and multivariate analysis of miR-148a expression and DFS in stage II/III CRC patients.

		II+III					III only		
			Univariate		Multivariate			Univariate	
Variables		n	HR (95%CI)	p	HR (95%CI)	p	n	HR (95%CI)	p
**Age, years**	>60	143	1.0		1.0		33	1.0	
	<60	58	1.57 (0.97–2.56)	0.071	1.78 (1.09–2.92)	0.022^b^	92	1.42 (0.79–2.56)	0.24
**Gender**	Male	118	1.0				78	1.0	
	Female	83	0.97 (0.60–1.57)	0.91			47	0.89 (0.50–1.60)	0.71
**Tumor location** a	Proximal	61	1.0				49	1.0	
	Distal	140	0.92 (0.55–1.51)	0.73			76	1.15 (0.64–2.07)	0.64
**MSI**	No	187	1.0				117	1.0	
	Yes	14	0.78 (0.29–2.13)	0.63			8	1.35 (0.49–3.73)	0.57
**Stage**	II	76	1.0		1.0				
	III	125	2.06 (1.21–3.52)	0.008^b^	2.06 (1.20–3.53)	0.009^b^			
**miR-148a expression**	High	138	1.0		1.0		80	1.0	
	Low	63	1.74 (1.08–2.83)	0.025^b^	1.83 (1.12–2.99)	0.017^b^	45	2.11 (1.21–3.68)	0.009^b^

a Proximal colon, located above splenic flexture; distal colon, located in splenic flexture or below.

b p<0.05.

We next analyzed data from stage II and III CRC separately to determine whether the association between low miR-148a expression and worse outcome was uniform across both stages, or predominantly aligned with one stage. We found that in stage II, miR-148a expression did not associate with DFS (5-year DFS, high vs. low, 77% vs. 83%, p = 0.50; **Figure 2C**) or OS (5-year OS, 89% vs. 87%, p = 0.94; data not shown). However, in stage III, low miR-148a expression was significantly associated with poorer DFS (5-year DFS, 43% vs. 66%, p = 0.0071; **Figure 2D**) but not with OS (5-year OS, 75% vs. 81%, p = 0.16; data not shown). Moreover, low miR-148a expression was an only factor that associated with tumor recurrence in stage III (**Table 2**). These results suggest that miR-148a expression status has a potential as a prognostic/predictive biomarker for stage III CRC.

### Low miR-148a expression is associated with worse a therapeutic response and worse survival in stage IV CRC

**Figure 3:**
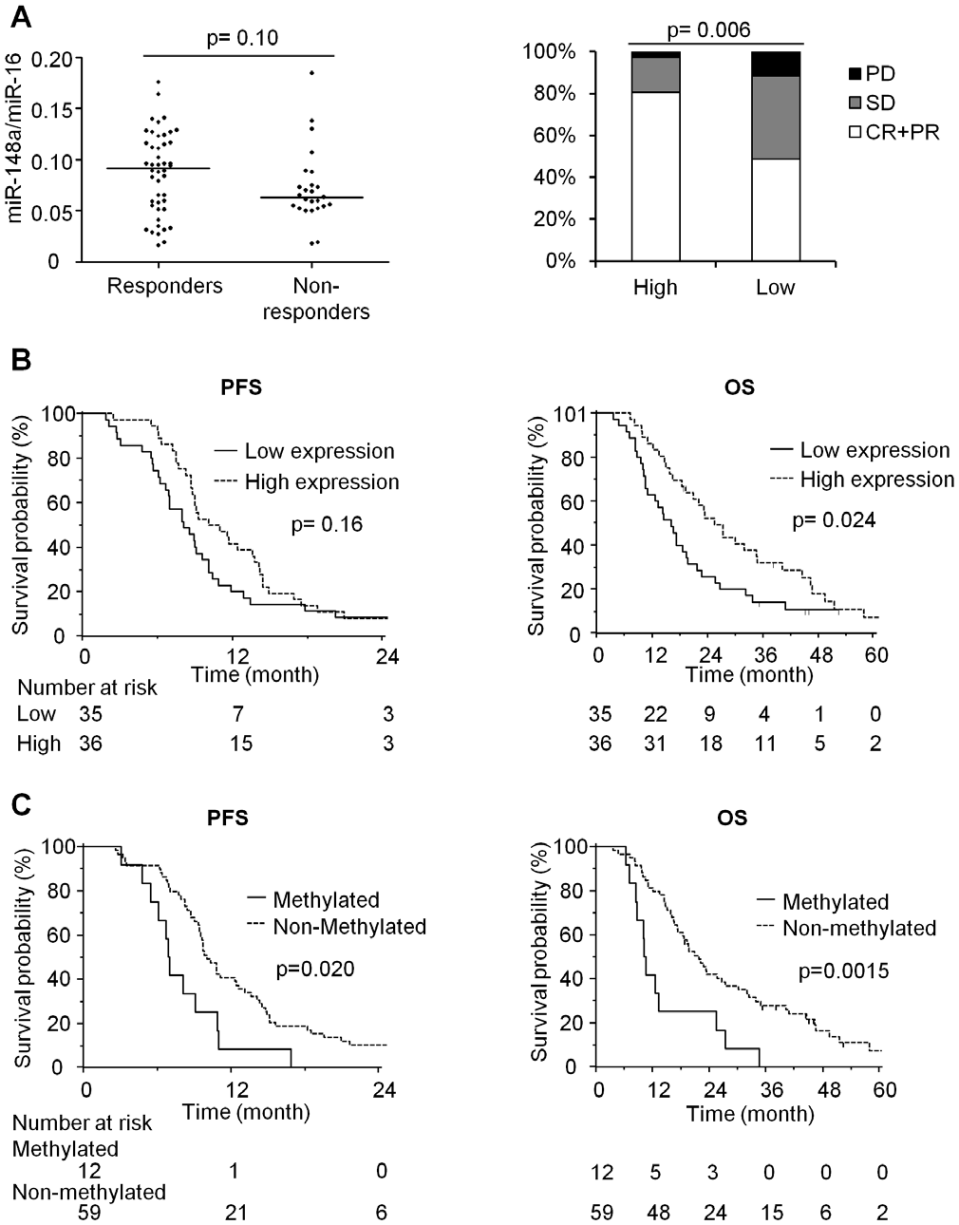
Associations between miR-148a status and therapeutic response or survival in stage IV CRC patients treated with 5-FU and oxaliplatin. **A**. Therapeutic response according to miR-148a expression. Complete response, CR; partial response, PR; stable disease, SD; progressive disease; PD. **B**. Kaplan-Meyer curves for progression-free survival (PFS, left panel) and OS (right panel) in stage IV patients according to miR-148a expression. **C**. Kaplan-Meyer curves for PFS (left panel) and OS (right panel) in stage IV patients according to miR-148a methylation.

We next elucidated whether miR-148a status had a potential for predicting therapeutic outcome in patients with stage IV CRC treated with 5-FU and oxaliplatin. Age, gender, tumor location, and performance status were not significantly different between the high and low expression groups (**Table 1**). Tumors from non-responders (stable disease and progressive disease) showed a trend toward lower miR-148a expression compared with those from responders (complete response and partial response) (median, 0.063 vs. 0.092, p = 0.10; **Figure 3A** **, left**). Nonetheless, when the stage IV tumors were divided into the low and high miR-148a expression groups, the low expression group was significantly associated with an unfavorable therapeutic response (responders, 49% vs. 81%, p = 0.006; **Figure 3A** **, right**). At Kaplan-Meyer analysis, the low expression group showed a trend toward worse PFS (median, 8.1 vs. 10.1 months, p = 0.16; **Figure 3B** **, left**) and significantly worse OS (16.1 vs. 25.6 months, p = 0.024; **Figure 3B** **, right**). In addition to the expression status, miR-148a methylation status also associated with both worse PFS (methylated vs. non-methylated, 6.9 vs. 9.3 months, p = 0.020; **Figure 3C** **, left**) and OS (10.2 vs. 21.8 months, p = 0.0015; **Figure 3C** **, right**).

We also evaluated the predictive value of miR-148a in a Cox proportional-hazard model. In univariate analysis, worse PS (HR 2.62, 95% CI 1.26–5.43, p = 0.010), lower miR-148a expression (HR 1.79, 95% CI 1.08–2.98, p = 0.026) and miR-148a hypermethylation (HR 2.76, 95% CI 1.44–5.28, p = 0.002) were significantly associated with worse survival (**Table 3**). In the final multivariate model that included these three factors, both miR-148a expression status (HR 1.93, 95% CI 1.15–3.23, p = 0.014) and miR-148a hypermethylation (HR 3.04, 95% CI 1.56–5.93, p = 0.0011) emerged as independent predictive factors that were associated with poorer outcome (**Table 3**).

**Table 3:** Univariate and multivariate analysis of miR-148a expression, methylation and overall survival in stage IV MSS CRC patients.

			Univariate		Multivariate	
		n	HR (95%CI)	p	HR (95%CI)	p
**Age (years)**	>60	35	1.0			
	<60	36	0.91 (0.55–1.50)	0.71		
**Gender**	Male	46	1.0			
	Female	25	0.68 (0.40–1.16)	0.16		
**Tumor location**	Colon	58	1.0			
	Rectum	13	1.41 (0.76–2.60)	0.27		
**Performance status**	0–1		1.0			
	2		2.62 (1.26–5.43)	0.010^a^		
**miR-148a expression**	High	36	1.0		1.0	
	Low	35	1.79 (1.08–2.98)	0.026^a^	1.93 (1.15–3.23)	0.014^a^
**miR-148a methylation**	No	59	1.0		1.0	
	Yes	12	2.76 (1.44–5.28)	0.002^a^	3.04 (1.56–5.93)	0.0011^a^

a p<0.05.

## Discussion

The aim of this study was to elucidate whether miR-148a expression is dysregulated in CRC, and furthermore, if this has the potential to serve as a prognostic or predictive biomarker in this disease. Herein, we provide evidence that highlight a significant role for miR-148a dysregulation in CRC. First, miR-148a expression was frequently down-regulated in CRC, particularly in advanced stage tumors. Second, miR-148a expression in colon was regulated at least in part through epigenetic mechanisms based upon the inverse correlation observed between its expression and methylation status. Third, low miR-148a expression was independently associated with poor prognosis in stage III patients treated with adjuvant chemotherapy. Fourth, low miR-148a expression was also associated with worse therapeutic response and poorer survival in stage IV patients treated with chemotherapy. Finally, the methylation status was an independent predictor for worse prognosis in stage IV CRC.

*In vitro* studies using non-colonic cell lines have indicated that miR-148a exerts a tumor suppressive function by targeting several genes such as *PXR*, *TGIF2*, *MSX1*, *CDC25B*, *DNMT1* and *DNMT3b* [39], [42]–[46]. The dysregulation of miR-148a has been implicated in CRC [14], [29], [39], [47], however, the clinical significance of altered miR-148a expression in CRC remains to be fully elucidated. In our study, we have analyzed the largest cohort of CRC patients to date (n = 273) and provided robust evidence that miR-148a expression is frequently down-regulated in advanced CRC and its reduced expression is associated with worse survival in stage III and IV disease. To the best of our knowledge, none of the previous reports have embarked upon the identification of miRNAs as biomarkers for predicting the therapeutic response in stage IV CRC patients treated with cytotoxic chemotherapy. Our most clinically important observation was that stage IV CRC patients with high miR-148a expression were more likely to benefit from cytotoxic chemotherapy, highlighting the potentially novel predictive value of this miRNA as a decision-making tool in the management of patients with CRC.

The precise mechanisms underlying miR-148a down-regulation in promoting resistance of CRC cells to chemotherapy require further elucidation. However, recent evidence for the role of this miRNA in other cancers have offered clues for understanding some of its effects on cellular chemosensitivity. Fujita *et al.* reported that miR-148a directly targets *MSK1* and the transfection of its precursor enhanced sensitivity to paclitaxel in prostate cancer cells [43]. miR-148a expression has also been shown to improve response to cisplatin and 5-FU in esophageal cancer cells [48]. In addition to these *in vitro* findings, in young patients with acute myeloid leukemia, higher *BAALC* gene expression correlates inversely with miR-148a expression, and has been shown to associate with worse outcome in patients treated with chemotherapy [49]. Collectively, our observations for the potential of miR-148a expression status as a predictive marker in stage IV CRC concur with these previous publications in other cancers.

Our study is also the first attempt to confirm methylation-mediated silencing of miR-148a by directly comparing expression and methylation levels in a large cohort of well-annotated CRCs. In addition to the initial studies by Lujambio *et al.* [39], more recently it was shown that miR-148a was hypermethylated in 51 out of 78 CRCs [40]. However, neither of these studies performed miR-148a expression analysis and directly correlated their results with hypermethylation in tissues. Furthermore, both studies analyzed miR-148a methylation status using a non-quantitative methylation-specific PCR method, which is notoriously non-specific for methylation, and does not provide a threshold for methylation that correlates with transcriptional inactivation of the gene. The strength of our study is that we determined miR-148a expression by qRT-PCR, and correlated the expression data with quantitative bisulfite pyrosequencing results, which is a more robust approach for demonstrating methylation-mediated dysregulation of any gene. Accordingly, we observed a significant inverse association between methylation and expression, reinforcing the concept that miR-148a down-regulation in CRC is due, in part, to promoter hypermethylation. We also noted a significant and independent association between miR-148a methylation and poor survival in stage IV patients, highlighting that expression and methylation status of miR-148a might be useful as prognostic/predictive markers in CRC. Finally, we confirmed our RT-PCR-based expression results by performing ISH on FFPE tissues, which allows a direct morphologic representation of the miRNA expression in the tissues. In these experiments we observed a significant correlation between qRT-PCR and ISH data, which suggests a potential translational application of ISH in clinical practice.

It should be noted that, our results in stage IV CRC implicates the predictive value of miR-148a expression status, however, it is still unclear that the miR-148a expression status has a prognostic, or predictive value, or both, in stage II and III CRC. In our study, all patients with stage II and III CRC were treated with 5-FU-based adjuvant chemotherapy, therefore, further cohorts of patients including both treated and non-treated with adjuvant chemotherapy are required to determine the prognostic and/or predictive value of the miR-148a expression status in stage II and III CRC.

In conclusion, our study describes the clinical significance of miR-148a in CRC, wherein we demonstrate that its expression is frequently down-regulated, particularly in advanced stage tumors. Furthermore, this study builds upon growing evidence that miRNA expression can be epigenetically regulated. Our data indicate that miR-148a expression, as well as its methylation status, may serve as predictive biomarkers in CRC. Our data provide a rationale for undertaking future studies to further validate the predictive value of miR-148a in the management of CRC patients treated with conventional chemotherapy and/or combinations of molecular-targeted drugs.

## Supporting Information

Table S1 **MiRNA expression analysis in a screening set by using multiplex realtime RT-PCR.**
(DOCX)
