# Supplementary material for: The Clinical Significance of MiR-148a as a Predictive Biomarker in Patients with Advanced Colorectal Cancer
Source: PLoS One. 2012 Oct 3;7(10):e46684. doi: 10.1371/journal.pone.0046684 (PMC3463512; doi:10.1371/journal.pone.0046684)
Supplement: Table S1 — MiRNA expression analysis in a screening set by using multiplex realtime RT-PCR. (DOCX) [file pone.0046684.s001.docx]

**Supplementary Table 1.** miRNA expression analysis in a screening set by using multiplex realtime RT-PCR

|  | **Mean expression normalized by miR-16** | | |  |  |
| --- | --- | --- | --- | --- | --- |
| **miRNA** | **Normal (n= 6)** | **Stage II (n= 16)** | **Stage III+IV (n= 28)** | **p^a^ (N vs II, III, or IV)** | **p^b^ (II vs III+ IV)** |
|  |  |  |  |  |  |
| miR-9 | N.D.^c^ | N.D.^c^ | N.D.^c^ |  |  |
| miR-10b | 0.12 | 0.028 | 0.035 | <0.001^d^ | 0.229 |
| miR-19a | 0.018 | 0.014 | 0.012 | 0.581 |  |
| miR-21 | 0.256 | 0.332 | 0.345 | 0.539 |  |
| miR-31 | 0.008 | 0.253 | 0.19 | 0.809 |  |
| miR-34a | 0.048 | 0.092 | 0.099 | 0.149 |  |
| miR-34c | N.D.^c^ | N.D.^c^ | N.D.^c^ |  |  |
| miR-101 | N.D.^c^ | N.D.^c^ | N.D.^c^ |  |  |
| miR-103 | 0.389 | 0.046 | 0.046 | <0.001^d^ | 0.996 |
| miR-137 | N.D.^c^ | N.D.^c^ | N.D.^c^ |  |  |
| miR-143 | 0.362 | 0.364 | 0.395 | 0.805 |  |
| miR-145 | 10.9 | 1.379 | 1.75 | <0.001^d^ | 0.285 |
| **miR-148a** | **0.077** | **0.043** | **0.026** | **<0.001^d^** | **0.016^d^** |
| miR-148b | N.D.^c^ | N.D.^c^ | N.D.^c^ |  |  |
| miR-152 | 0.038 | 0.052 | 0.058 | 0.72 |  |
| miR-155 | 0.916 | 0.327 | 0.418 | 0.012^d^ | 0.161 |
| miR-194 | 0.359 | 0.173 | 0.143 | <0.001^d^ | 0.249 |
| miR-320 | 2.347 | 1.091 | 1.176 | 0.040^d^ | 0.582 |
| miR-355 | N.D.^c^ | N.D.^c^ | N.D.^c^ |  |  |
| miR-373 | N.D.^c^ | N.D.^c^ | N.D.^c^ |  |  |
| miR-519c | N.D.^c^ | N.D.^c^ | N.D.^c^ |  |  |
| ^a^determined by ANOVA and post-hoc test. | | |  |  |  |
| ^b^determined by Student's-t test. | | |  |  |  |
| ^c^not determined. | |  |  |  |  |
| ^d^p<0.05 |  |  |  |  |  |
